# Supplementary material for: Less Is More: Selective-Atom-Removal-Derived Defective MnOx Catalyst for Efficient Propane Oxidation
Source: Nanomaterials (Basel). 2024 May 22;14(11):907. doi: 10.3390/nano14110907 (PMC11173853; doi:10.3390/nano14110907)
Supplement: Supplementary file 1 [file nanomaterials-14-00907-s001.zip › nanomaterials-2990076-supplementary.pdf]

nanomaterials

Supporting Materials

Less Is More: Selective-Atom-Removal-Derived Defective MnO_x Catalyst for Efficient Propane Oxidation

Wenfan Xu [1,†], Limei Zhou [2,†], Lining Liu [1], Huimei Duan [3,*], Haoxi Ben [1,*], Sheng Chen [4] and Xingyun Li [1,*]

1 State Key Laboratory of BioFibers and Eco-Textiles, Institute of Materials for Energy and Environment, College of Materials Science and Engineering, Qingdao University, Qingdao 266071, China; yanwu003x@foxmail.com (W.X.); lln2584038492@163.com (L.L.)
2 Chemical Synthesis and Pollution Control Key Laboratory of Sichuan Province, China West Normal University, Nanchong 637002, China; cwnuzhoulimei@163.com
3 Institute of Marine Biobased Materials, School of Environmental Science and Engineering, Qingdao University, Qingdao 266071, China
4 State Key Laboratory of Coal Combustion, School of Energy and Power Engineering, Huazhong University of Science and Technology, Wuhan 430074, China; sheng_chen@hust.edu.cn
* Correspondence: duanhm@qdu.edu.cn (H.D.); benhaoxi@qdu.edu.cn (H.B.); xingyun_2008@sina.cn (X.L.)
† These authors contribute equally to this work.

Text S1 Catalysts characterizations

Powder X-ray diffraction (XRD) was performed on a Rigaku Ultima IV with Cu Kα (λ = 0.154178 nm) as radiation source. N_2 adsorption-desorption experiment was carried out by Quantachrome Autosorb iQ3 at 77 K. Before measurement, catalysts were degassed at 200 °C for 10 h to expel the adsorptions. Brunauer-Emmett-Teller (BET) model was adapted to calculate the specific surface area and Barrett-Joyner-Halenda Method (BJH) was used to evaluate the pore size distribution. The field emission scanning electron microscopy (FE-SEM) and energy dispersive spectrometer (EDS) was examined by JEOL JSM-7800F. The transmission electron microscopy (TEM) was recorded on JEOL JEM-2100. X-ray photoelectron spectroscopy (XPS) was recorded on PHI 5000 Versa Probe III using an Al Kα radiation source (1486.6 eV). Raman scattering spectra were performed on Renishaw in Via Plus Micro-Raman spectroscopy with a 532 nm wavelength provided by 50 mW DPSS laser. Inductively coupled plasma (ICP) was obtained by Agilent ICP-OES/730. Electron paramagnetic resonance (EPR) was tested by FA-200 (JES) electron paramagnetic resonance spectrometer. Hydrogen temperature programmed reduction (H_2-TPR) was measured on a Quantachrome chamber pulsar analyzer. Specifically, catalysts were pretreated in He at 200 °C for 1 h, and then cooled to room temperature. Then, the catalysts was heated from 50 °C to 600 °C (10 °C·min^{-1}) in 5% H_2 (Ar balanced) atmosphere (100 mL·min^{-1}). The signal was recorded by an online gas mass spectrometer (Hiden, HPR-20 EGA). *In-situ* diffuse reflectance infrared Fourier transform spectroscopy (*In-situ* DRIFTS) was measured on a Bruker Equinox 55 infrared analyzer equipped with an MCT/A detector and an in-situ cell. Before each experiment, the samples were dried at 80 °C for 6 h and cooled to room temperature. Degassing was first carried out at 300 °C for 1 h under Ar atmosphere. After cooling to room temperature, the background was collected in the Ar flow, then the gas was changed to to 0.5 *vol.*% C_3H_8, 10 *vol.*% O_2, balanced with Ar (25 mL·min^{-1}). Data were collected under different temperatures (50 °C, 100 °C, 150 °C, 200 °C, 250 °C, 300 °C). Infrared spectral data of the samples were acquired by accumulating 64 scans with a resolution of 8 μm^{-1}.

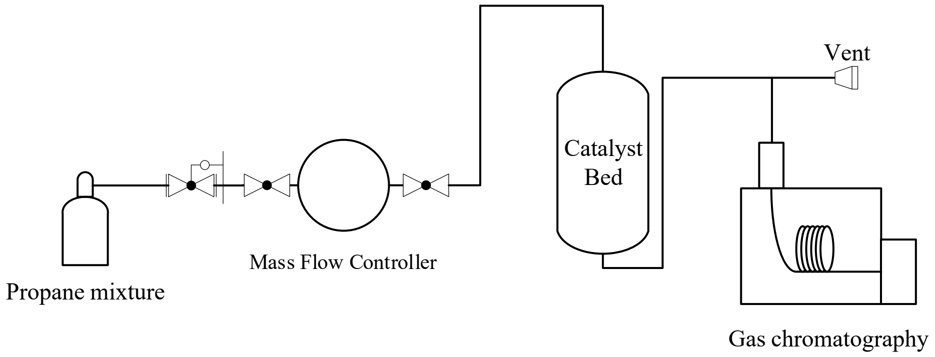

Figure S1. Set-up diagram of propane catalytic oxidation.

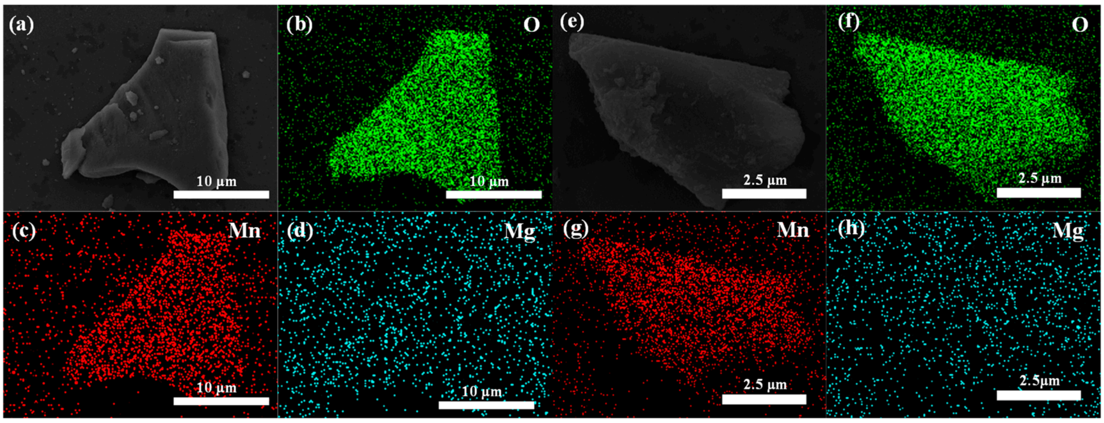

Figure S2. (a, e) SEM image and EDS elemental mapping (b, f) O, (c, g) Mn and (d, h) Mg of MgMnOx and MgMnOx-H.

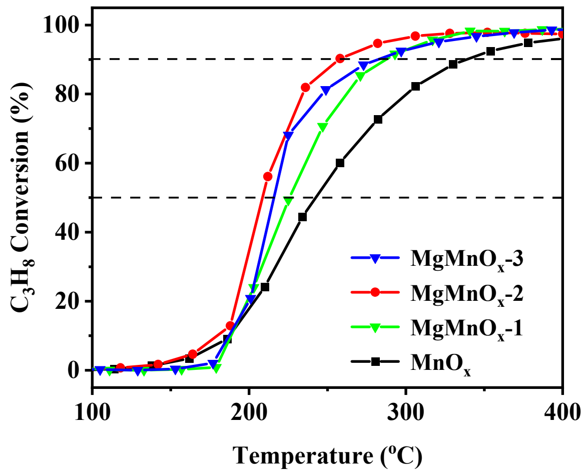

Figure S3. Catalytic performance of MnO_x, $MgMnO_x$-1, $MgMnO_x$-2 and $MgMnO_x$-3.

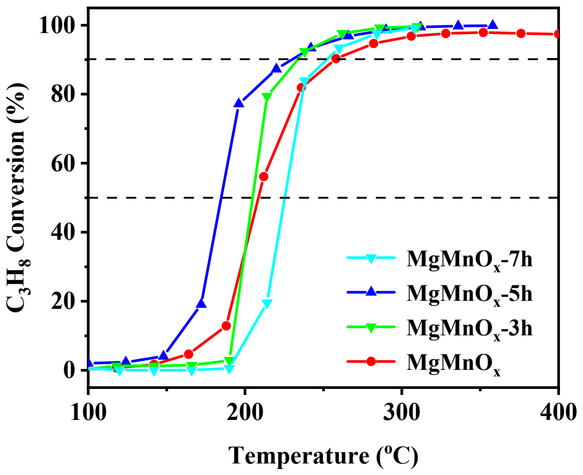

Figure S4. Catalytic performance of MgMnOx, MgMnOx-3h, MgMnOx-5h, MgMnOx-7h.

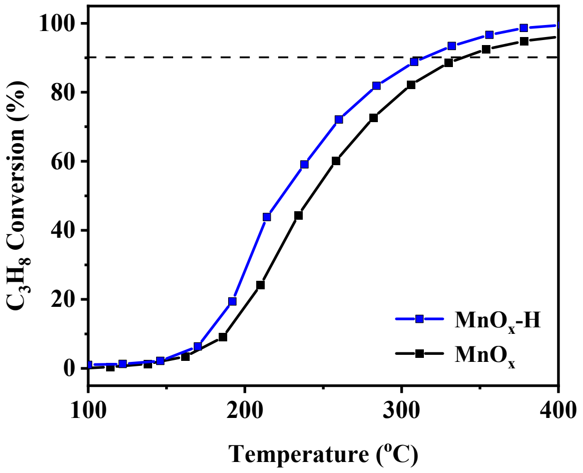

Figure S5. Catalytic performance of MnO_x and MnO_x-H.

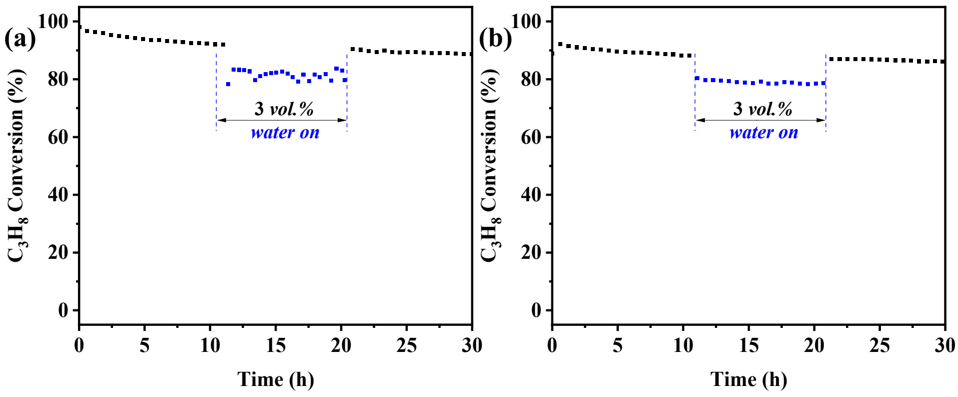

Figure S6. Hydrothermal stability test of (a) MnO_x and (b) $MgMnO_x$ at temperature corresponding to propane conversion above 90% (Reaction conditions: 0.5 *vol.*% C_3H_8, 10 *vol.*% O_2, Ar as balance gas, GHSV = 60,000 $mL{\cdot}g^{-1}{\cdot}h^{-1}$).

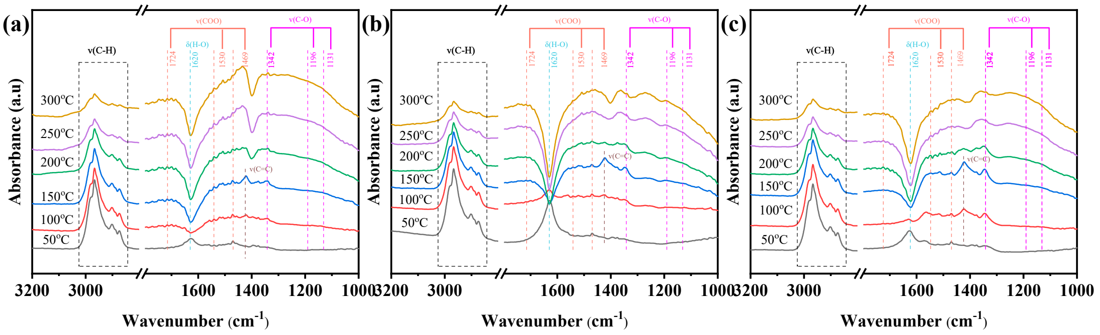

Figure S7. *In-situ* DRIFTS spectra of propane oxidation (0.5% C_3H_8, 10% O_2, balanced with N_2) on (a) MnO_x, (b) $MgMnO_x$ and (c) $MgMnO_x$-H.
